# *MSCTrees*: a mean-shift based toolkit for cluster analysis of phylogenetic trees

**DOI:** 10.1186/1471-2105-12-S7-A13

**Published:** 2011-08-05

**Authors:** Weixi Li, Jerzy W Jaromczyk

**Affiliations:** 1 Department of Computer Science, University of Kentucky, Lexington, KY, 40506, USA; 2 Department of Biology, University of Kentucky, Lexington, KY, 40506, USA

## Background

Mean shift, an iterative technique for identifying the local maxima of a probability density function, has been successfully used as a clustering method in computer vision and image processing. We apply the mean shift technique to the high dimensional space of phylogeny trees. The basic idea behind this technique is to, given a set of sample points, *shift* each point in the direction of the gradient of the underlying density function in an iterative manner until the points concentrate at the local maxima of the density function and form natural clusters [1]. We have developed software named *MSCTrees* based on a variant of the mean shift method, called the adaptive mean shift [2], to perform cluster analysis on a set of multidimensional data points corresponding to phylogenetic trees.

## Methods

*MSCTrees* has two components: a C program called *ms_cluster* which implements a clustering algorithm based on the adaptive mean shift method, and a Perl script called *cluster_trees.pl*, which converts phylogenetic trees to multidimensional data points and calls *ms_cluster* to perform cluster analysis on the resulting points. The *ms_cluster* program, developed in C for optimized performance, takes a set of multidimensional data points as input, and outputs the clusters of the input points together with the cluster centers. The *ms_cluster* performs the following steps: 1) calculate the adaptive bandwidth for each data point using the *k*-Nearest Neighbor (*k*NN) method; 2) initialize a set of points using the values of the original data points; 3) shift the set of initialized points to new locations based on the mean shift vectors computed at each point; 4) repeat step (3) until all points have converged; 5) merge points that have converged to the same locations into clusters. Four auto-optimized (and user-definable) parameters have been implemented to control the mean shift clustering process.

The *cluster_trees.pl* script uses the BioPerl modules to parse a set of phylogenetic trees as the input. It maps a phylogenetic tree to a multidimensional data point by calculating the pair-wise distances between the leaves of the tree as the dimensional values of the resulting point. The script produces as output clusters of phylogenetic trees resulting from the clustering of their corresponding data points.

## Results and conclusion

We tested *MSCTrees* with a well-known gopher-louse data set, which contains two sets of phylogenetic trees (101 trees each) for 15 species of gophers and 15 species of lice, respectively. Separate cluster analyses were performed on the two sets of trees, followed by a cluster analysis on the combined tree sets. A significantly reduced number of clusters was obtained on the combined data, which suggests similarity between the two tree distributions and is consistent with the known co-evolutionary relationship between gophers and lice. The pilot results demonstrate that the *MSCTrees* tool has strong potential for effective high dimensional cluster analysis of phylogenetic trees. We are also investigating other phylogenetic applications, such as identifying gene transfers via outlier detection.
